# A centroid iterative method for fitting the femoral neck axis in rotational osteotomy: a finite element analysis and biomechanical investigation

**DOI:** 10.1093/jhps/hnaf026

**Published:** 2025-05-08

**Authors:** Liang Chen, Zexin Hong, Zhentao Ding, Jingyang Chen, Zhuohua Liu, Chenglin Chen, Yufeng Wu, Jianhai Chen, Dawei Gao

**Affiliations:** Department of Orthopaedic Surgery, Traditional Chinese Medicine Hospital of Zhongshan, Guangzhou University of Chinese Medicine, 3 Kangxin Road, Guangdong, Zhongshan 528401, China; Department of Orthopaedic Surgery, Traditional Chinese Medicine Hospital of Zhongshan, Guangzhou University of Chinese Medicine, 3 Kangxin Road, Guangdong, Zhongshan 528401, China; Department of Orthopedics and Trauma, Peking University People’s Hospital, 11 South Xizhimen Street, Beijing 100044, China; National Center for Trauma Medicine, Peking University People’s Hospital, 11 South Xizhimen Street, Beijing 100044, China; Key Laboratory of Trauma and Neural Regeneration (Peking University), Ministry of Education, 11 South Xizhimen Street, Beijing 100044, China; Department of Orthopaedic Surgery, Traditional Chinese Medicine Hospital of Zhongshan, Guangzhou University of Chinese Medicine, 3 Kangxin Road, Guangdong, Zhongshan 528401, China; Department of Orthopaedic Surgery, Traditional Chinese Medicine Hospital of Zhongshan, Guangzhou University of Chinese Medicine, 3 Kangxin Road, Guangdong, Zhongshan 528401, China; Department of Orthopaedic Surgery, Traditional Chinese Medicine Hospital of Zhongshan, Guangzhou University of Chinese Medicine, 3 Kangxin Road, Guangdong, Zhongshan 528401, China; Department of Orthopaedic Surgery, Traditional Chinese Medicine Hospital of Zhongshan, Guangzhou University of Chinese Medicine, 3 Kangxin Road, Guangdong, Zhongshan 528401, China; Department of Orthopedics and Trauma, Peking University People’s Hospital, 11 South Xizhimen Street, Beijing 100044, China; National Center for Trauma Medicine, Peking University People’s Hospital, 11 South Xizhimen Street, Beijing 100044, China; Key Laboratory of Trauma and Neural Regeneration (Peking University), Ministry of Education, 11 South Xizhimen Street, Beijing 100044, China; Department of Orthopaedic Surgery, Traditional Chinese Medicine Hospital of Zhongshan, Guangzhou University of Chinese Medicine, 3 Kangxin Road, Guangdong, Zhongshan 528401, China

## Abstract

The femoral neck axis (FNA) is an important reference in femoral neck rotational osteotomy, which is a hip preservation procedure. The purpose of this study was to propose a method for determining the FNA with high accuracy and reliability and to evaluate the effect of FNA determination accuracy on the stress and strain distribution in the proximal femur. Femoral computed tomography data from 50 patients were reconstructed, and the FNA was fitted by the centroid iterative method. The fitting accuracy was evaluated in terms of the distance between the femoral head centre and the FNA. The reliability was assessed by intraclass correlation coefficient (ICC). Stress–strain distributions of the native femur model and rotational osteotomy models with accurate FNA and deviated FNA were simulated by finite element analysis and digital image correlation methods. The distance between the femoral head centre and the FNA was 1.24 ± 0.35 mm. The intra- and interobserver reliability was high, with ICC values of 0.960 and 0.924, respectively. The maximum von Mises stress was 25.76 MPa, 50.82 MPa, and 93.24 MPa for the native femur model, accurate FNA model, and deviated FNA model, respectively. The finite element strain distributions were linearly correlated with digital image correlation results. The centroid iterative method for FNA fitting has high accuracy and reliability. Accurate determination of the FNA reduces stress concentration in the proximal femur and decreases the risk of subsequent fracture and non-union of the osteotomy surface.

## Introduction

Young patients with femoral head necrosis suffer from poor hip prosthesis survivorship [1]. In the early stage of femoral head necrosis, hip preservation surgeries have attracted extensive attention as they are able to postpone hip replacement [2, 3]. Femoral neck rotational osteotomy is a surgical procedure to preserve the femoral head. After osteotomy at the base of the femoral neck, the necrotic area of femoral head is rotated 60°–90° forward or backward to the non-weight-bearing area of the hip joint to prevent further collapse [4]. Intraoperatively, the Kirschner wire implanted along the femoral neck axis (FNA) should be as precise as possible to avoid variations in the anteversion angle and neck-shaft angle after rotation [5]. In practice, however, the determination of FNA is based on the surgeon’s experience, and deviations in the determination can lead to complications such as subsequent collapse and progressive osteoarthritis [6, 7]. Therefore, the determination of FNA needs further optimization.

The FNA is difficult to determine accurately by direct computed tomography (CT) images, and the centroid iterative method is a new strategy for FNA fitting [8]. Mathematically, the femoral neck region can be considered as an integral set of centroid point clouds of multiple sections. Based on this, an axis is fitted to finite centroids through the least squares method. After several iterations of calculation, until the results converge, the final fitted axis is considered to be the FNA [9].

Finite element analysis (FEA) is a non-invasive biomechanical simulation method with many advantages over cadaveric specimens [10, 11]. Digital image correlation (DIC) is a new technique for measuring full-field strain that generates accurate two-dimensional strain maps [12–14]. However, no studies have been seen on these methods applied to the FNA and rotational osteotomy.

The objectives of this study were as follows: (1) to investigate the accuracy and reliability of fitting FNA by centroid iterative method and (2) to compare the stress–strain distribution of the proximal femur based on rotational osteotomy models simulated by accurate FNA and deviated FNA.

## Materials and methods

### Patients

A total of 50 patients were included by collecting bilateral femoral CT data in our institution from January 2019 to January 2020. This included 27 males and 23 females, aged between 45 and 78 years. The inclusion criteria were as follows: (1) patients with unilateral femoral head necrosis classified as Association Research Circulation Osseous stage IIB–IIIC; (2) intact femoral head with adequate range of non-necrotic areas within; (3) no severe femoral deformity; (4) no history of proximal femoral surgery; and (5) no history of rheumatoid arthritis.

### Femoral neck axis fitting

All patients underwent bilateral femoral CT scans (Philips, Holland) in same parameters with the slice thickness of 1 mm. Image data were imported into Mimics 21 (Materialise, Belgium), and 3D models of the proximal femur were reconstructed based on grey-scale values of the tissue and segmentation of the thresholds. The model data were imported into Geomagic Studio 2012 (3D Systems, USA) in STL format.

**Figure 1.**
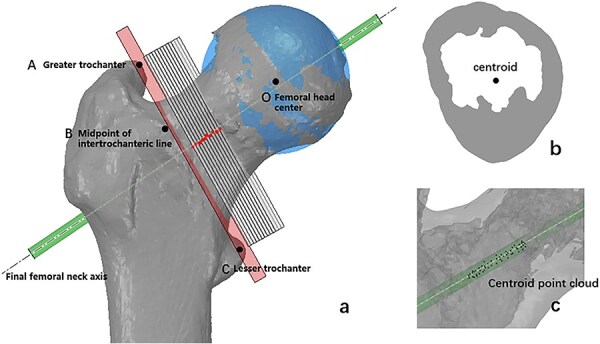
Centroid iterative method for fitting femoral neck axis. (a) The tip of the greater trochanter, the tip of the lesser trochanter, and the midpoint of the intertrochanteric line formed the initial section (red section). A total of 10 sections with 1 mm distance (grey sections) were obtained by isometric translation of the initial section. (b) The polygon algorithm was used to calculate the centroid of each section. (c) After five iterations of calculation, the centroid point cloud of all sections was cylindrically fitted. The axis of the cylinder was considered the final FNA.

The plane formed by the tip of the greater trochanter, the tip of the lesser trochanter, and the midpoint of the intertrochanteric line was set as the initial section (Fig. 1a). A total of 10 sections were obtained by isometric translation of the initial section along the femoral neck, each with the distance of 1 mm. The centroid for each section was calculated using the polygonal centroid algorithm (Fig. 1b) [15]. A new axis was then obtained by fitting a straight line to the centroid point cloud of isometric sections by the least squares method. The isometric sections were constructed again perpendicular to the new axis. The above method was repeated, and the cylindrical fitting was performed for the centroid point cloud of all sections after five iterations of calculation. The axis of the cylinder was considered the final FNA (Fig. 1c).

The femoral head could be fitted as a sphere to obtain a spherical centre [16]. To ensure the mechanical equilibrium of the proximal femur, it was assumed that the ideally fitted spherical centre was located on the FNA [17]. The distance between the femoral head centre and the FNA was measured and applied to evaluate the accuracy of FNA fitting.

### Finite element models

**Figure 2.**
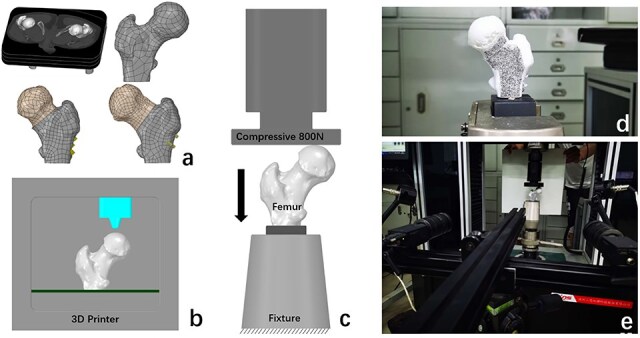
Finite element analysis and mechanical testing. (a) CT data were used to construct finite element models for normal, accurate, and deviation cases. (b) A 3D printer was applied to fabricate the femur models for mechanical testing. (c) The same loading conditions were used for FEA and mechanical testing. (d) Scatter fabrication of the femoral neck surface. (e) The strain field was measured by the DIC method during the mechanical testing.

A bilateral femoral 3D model was selected for inverse design, and the NURBS CAD model was obtained by meshing and surface fitting. Three different loading cases were simulated for comparison, including normal, accurate, and deviation cases, to evaluate the effect of FNA fitting accuracy on the stress–strain distribution in the proximal femur (Fig. 2a). The normal case was modelled based on the healthy femur, and the accurate and deviation cases were modelled based on the affected femur. In the accurate case, the FNA was fitted by the centroid iterative method. The osteotomy was performed at the base of the femoral neck perpendicular to the FNA, and the necrotic part of femoral head was rotated around the axis to the non-weight-bearing area of the hip joint. In the deviation case, the FNA was rotated 20° clockwise, and the osteotomy direction was rotated accordingly. The femoral head was rotated around the new axis by the same angle.

A 7.3 mm diameter screw model was designed by Solidworks (Dassault Systemes, France). Three screws were implanted parallel to the FNA in the femur model for accurate and deviation cases, respectively. The models were imported into ANSYS (ANSYS Inc., USA) for meshing, and the element type was an unstructured linear triangle. The mesh convergence analysis was completed.

### Boundary and loading conditions

All materials were assumed to be isotropic, homogeneous, and linearly elastic [18]. The elastic modulus and Poisson’s ratio of the materials are shown in Table 1. For accurate and deviation models, contact friction was defined between the osteotomy surfaces and between the screw and the femur, both with a friction coefficient of 0.2. A vertical compressive load of 800 N was applied to the femoral head [19]. The distal femur was constrained to a mobility of 0°–6° (Fig. 2c). After performing the nonlinear analysis, the stress–strain distribution in the femoral neck region was compared among the three finite element models.

**Table 1.** Mechanical properties of various materials

Material	Elastic modulus (MPa)	Poisson’s ratio
Cortical bone	16 800	0.3
Cancellous bone	840	0.2
Screw	20 600	0.3

### Mechanical testing

Normal, accurate, and deviation models were input into a 3D printer (UnionTech Ltd, China) to fabricate the femur models for mechanical testing (Fig. 2b). The printer featured stereolithography appearance technology and medical-grade photoreceptor resin (Somos D638-14). To resemble the human bone, the composite bone model consists of cortical bone (elastic modulus of 2310 MPa) and cancellous bone injected with rigid polyurethane (elastic modulus of 155 MPa). The same screw fixation method as in the finite element models was used for the accurate and deviation cases.

The distal femur was fixed to the baseplate. The anterior surface of the composite bone was coated with DIC spray paint in a white background colour and a black random speckle pattern at the femoral neck (Fig. 2d). The DIC system was calibrated with a test block prior to mechanical testing. 3D printed models were loaded in a servo-hydraulic testing machine (Sunstest, China). The same loading conditions as FEA were applied, with a load range of 0–800 N (Fig. 2c). During the mechanical testing, a 0.2-megapixel digital camera (Pentax, Japan) was used to record the scatter displacements due to loading action and to acquire images. The strain field was subsequently analysed by the DIC method (Fig. 2e).

### Statistical analysis

Two-way analysis of variance (ANOVA) was performed to compare differences in the distance between the femoral head centre and the FNA by gender and side [20]. Reliability of the fitting method was assessed by intraclass correlation coefficient (ICC) [21]. Based on CT images of 50 patients, one investigator measured the random 10-case sample, counting each case three times, more than 24 hours apart, to assess intraobserver reliability. Different investigators measured the 10-case sample in an independent manner and in random order to assess inter-observer reliability. Linear regression analysis was performed on the strain values of FEA and DIC. The data were statistically analysed by SPSS 21.0 software (IBM, USA). The significance level was set at .05.

## Results

### Femoral neck axis

The distance between the femoral head centre and the FNA was 1.24 ± 0.35 mm. The statistical results were not significantly different between genders (*P* = .769), between sides (*P* = .107), and between the interaction of gender and side (*P* = 1.000) (Table 2). The intra- and interobserver reliability was high, with ICC values of 0.960 and 0.924, respectively (Table 3).

**Table 2.** Differences in the distance between head centre and femoral neck axis (mean ± SD) by gender and side

	Males (27)		Females (23)				
	Left	Right	Left	Right	*P*_1_	*P*_2_	*P*_3_
DBHF	1.19 ± 0.33	1.25 ± 0.36	1.16 ± 0.32	1.32 ± 0.35	.769	.107	1.000

DBHF, distance between head centre and femoral neck axis.

*P*_1_ for the effect of gender, *P*_2_ for the effect of side, and *P*_3_ for the interaction between gender and side, two-way ANOVA.

**Table 3.** Intraobserver and interobserver reliability of the measurements.

	Intraobserver reliability		Interobserver reliability	
DBHF	ICC	95% CI	ICC	95% CI
	0.960	0.920–0.983	0.924	0.850–0.967

ICC, intraclass correlation coefficient.

### Finite element analysis

**Figure 3.**
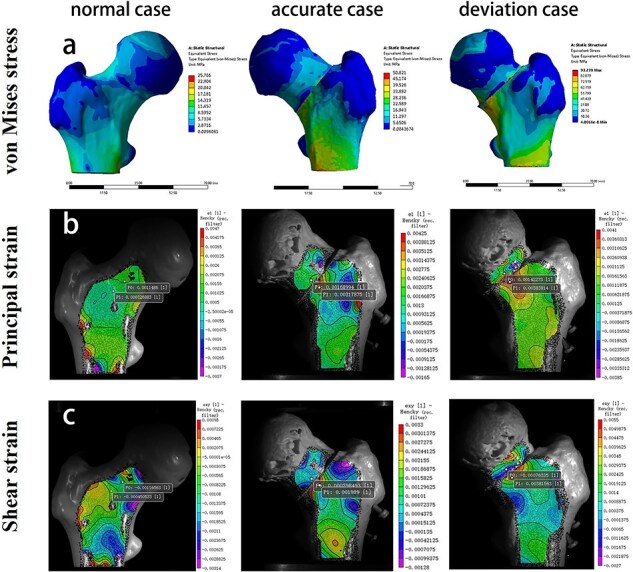
(a) The von Mises stress cloud charts of the proximal femur in finite element analysis. Principal strain (b) and shear strain (c) cloud charts in mechanical testing measured by the DIC method.

**Figure 4.**
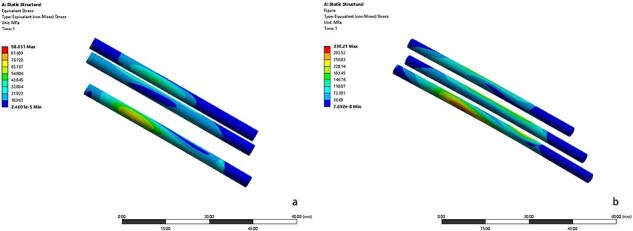
The von Mises stress cloud charts of screw models in the accurate case (a) and deviation case (b).

The maximum von Mises stress in the femoral neck region was 25.76 MPa, 50.82 MPa, and 93.24 MPa in normal, accurate, and deviation cases, respectively (Fig. 3a), and the maximum principal strain was 0.19 mm, 0.28 mm, and 0.47 mm, respectively. The maximum von Mises stress of screws was 98.65 MPa in the accurate model and 330.21 MPa in the deviation model. (Fig. 4). The stress concentration was clearly found at the tail end of screws.

### Mechanical testing

At the macroscopic scale, the stress distribution patterns of FEA were similar to the DIC strain field in the femoral neck region for the three models (Fig. 3). The maximum principal strain measured by the DIC method was 0.21 mm, 0.32 mm, and 0.46 mm for normal, accurate, and deviation cases, respectively. Regression analysis showed that the finite element strain distributions in normal, accurate, and deviation cases were linearly correlated with the DIC results. The correlation coefficients were high (*r*_1_ = 0.97, *r*_2_ = 0.98, and *r*_3_ = 0.96), and the regression coefficients were all 1.03.

**Figure 5.**
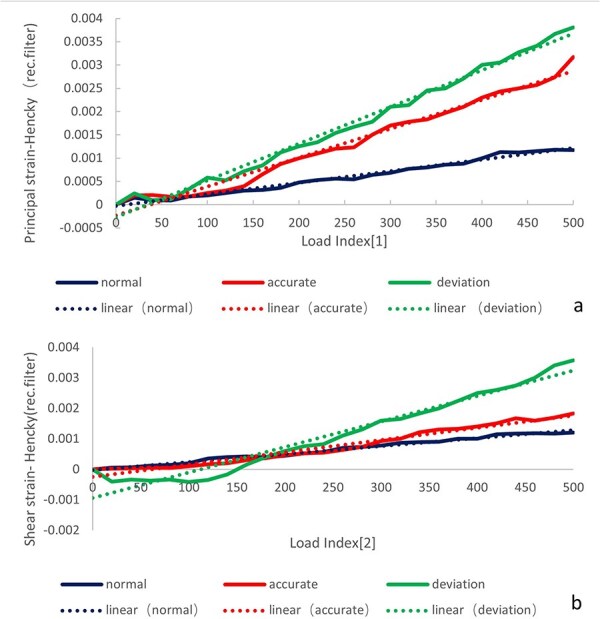
Load–strain curves for normal, accurate, and deviation cases. (a) Load–principal strain curve. (b) Load–shear strain curve.

The DIC results of principal strain and shear strain in the femoral neck region were shown in Fig. 3b and c. The 3D printed models were under elastic deformation in the load range of 0–500 N. The load–strain curves of principal strain and shear strain are shown in Fig. 5. The overall stiffnesses of normal and accurate models were similar. The deviation model had the largest variation in overall stiffness.

## Discussion

In this study, the centroid iterative method was found to fit the FNA with high accuracy and reliability. The results of FEA and DIC consistently suggested that accurate determination of the FNA could reduce the stress concentration in the proximal femur.

Sugioka *et al*. first proposed transtrochanteric rotational osteotomy for the treatment of femoral head necrosis in young patients in 1978 [22]. In long-term follow-up, approximately 78%–86% survival of the femoral head was observed [23]. However, related studies in Europe and the USA reported a high incidence of complications, including femoral head collapse, progressive osteoarthritis, subsequent fractures, and non-union of the osteotomy surface [6].

As an important reference for rotational osteotomy, there is no gold standard for the determination of FNA. Earlier, the FNA was a two-dimensional axis determined by X-ray or two-dimensional CT scan [24, 25]. Nakanishi *et al*. and Yin *et al*. identified the femoral head and neck on the coronal plane of three-dimensional CT images, defining the line between the femoral head centre and the neck isthmus centre as the FNA [26, 27]. However, both methods are influenced by the femur position during fluoroscopy. Zhang *et al*. performed a spherical fitting to the femoral head, obtaining the section by tangenting the offset sphere to the femoral isthmus [28]. An ellipse fitting was then performed based on its contour and the line passing through the ellipse centre, and the femoral head centre was considered as the FNA. However, the femoral neck section is not a standard ellipse, and it is not possible to fit the spherical centre accurately in some patients with femoral head collapse. Therefore, the fitting error of this method is relatively high. Masjedi *et al*. proposed a centroid iterative fitting method based on the femoral neck section [8]. But at least 15 iterations are required to achieve convergence, and the fitting process is too tedious for clinical application.

In this study, the previous methods were modified to exclude the influence of femoral spatial position by 3D reconstruction, and FNA fitting was performed by the centroid iterative method. The final FNA was 1.24 ± 0.35 mm from the femoral head centre, which provided high accuracy along with high reliability. The current controversy is the relative spatial position of the FNA to the femoral head centre. Pauwels *et al*. assumed that the femoral head centre is located on the FNA according to mechanical equilibrium [17]. However, Kingsley and Olmsted’s research did not support this assumption with a distance deviation of about 1 mm [29]. Therefore, a distance of 1.24 mm can be considered accurate and acceptable.

This study compared the stress–strain distribution of the proximal femur in normal, accurate, and deviation cases by FEA and DIC methods, and consistent results were obtained. In the normal case, the stress was distributed uniformly and continuously along the medial region of the femoral neck. This may be due to the role of compression trabeculae in transmitting and dispersing stress. In both osteotomy models, significant stress concentration was observed around the osteotomy surface. In the deviation case, the maximum von Mises stress was significantly higher in both the bone and screw models than in the accurate case, indicating that the deviation of FNA could exacerbate the stress concentration. The stress concentration is an important cause of subsequent fracture and non-union of the osteotomy surface after rotational osteotomy [30]. This suggests the importance of accurate determination for FNA.

There are limitations in this study. The CT resolution affects the fineness of finite element models. The FNA fitting is based on the centroid of isodensity femoral neck sections, and there is a certain fitting error with the actual FNA. The 3D printed models are difficult to simulate the isotropic and non-homogeneous properties of the human femur. Moreover, the surgical procedure may cause greater errors compared to the FNA fitting method. Accurate FNA is not the most critical requirement in clinical practice, but it is the only source of error that can be optimized during the preoperative planning. This study did evaluate the accuracy and reliability of the centroid iterative method for FNA fitting, which is expected to provide theoretical support for the FNA determination method. Intraoperative navigation is probably the key approach to transfer these results clinically. Based on this method, intraoperative navigation allows the practical application of preoperatively fitted FNA in orthopaedic surgery, achieving precise and individualized osteotomy at the base of femoral neck, while also minimizing errors due to surgical procedures.

## Conclusion

This study provided an accurate and reliable method for fitting the FNA to ensure the safety and reproducibility of rotational osteotomy. The deviations in FNA determination that led to stress concentration in the proximal femur were analysed by FEA and DIC. Although more clinical data and further biomechanical studies are needed in the future, this study provides important methodological support for the determination of FNA.

## Data Availability

The datasets and materials are available from corresponding authors on reasonable request.
